# From Brain to Behavior: Hypertension's Modulation of Cognition and Affect

**DOI:** 10.1155/2012/701385

**Published:** 2012-02-19

**Authors:** J. Richard Jennings, Alicia F. Heim

**Affiliations:** Departments of Psychiatry and Psychology, University of Pittsburgh, Pittsburgh, PA 15213, USA

## Abstract

Accumulating evidence from animal models and human studies of essential hypertension suggest that brain regulation of the vasculature is impacted by the disease. Human neuroimaging findings suggest that the brain may be an early target of the disease. This observation reinforces earlier research suggesting that psychological factors may be one of the many contributory factors to the initiation of the disease. Alternatively or in addition, initial blood pressure increases may impact cognitive and/or affective function. Evidence for an impact of blood pressure on the perception and experience of affect is reviewed vis-a-vis brain imaging findings suggesting that such involvement in hypertensive individuals is likely.

## 1. Introduction

Despite a long history of putative relationship between essential hypertension and psychological function, clear, particularly mechanistic, relationships remain elusive. Early work in humans postulated relationships between anger regulation and blood pressure [1] and work in animal models has long shown brain involvement in heightening blood pressure [2–4]. Recent animal investigations have reinforced this relationship and further implicated immune influences on blood pressure control [5]. The advent of brain imaging has been useful in specifying more clearly the neural involvement in hypertension and providing a possible bridge between behavioral and self-report information in humans and the mechanistic observations in animal models. The initial focus of the current paper is on neuroimaging results in humans. We argue that these findings suggest that the brain is an early target for hypertension. We then explore the implications for cognition and affect of these brain findings. Ideally, brain imaging can be used to further not only assessment of brain mechanisms but also complement psychological and neurophysiological investigations of the widely prevalent disease of essential hypertension.

## 2. Cross-Sectional Findings

Our work has led us to raise the possibility that essential hypertension early in its course alters brain function, see recent summary [6]. Initial studies compared normotensive individuals with hypertensive individuals without recent history of medication treatment [7, 8]. Based on prior work showing mild cognitive deficits in hypertensive individuals [9], we examined brain function using positron emission tomography while participants performed a working memory task; brain structure was assessed using magnetic resonance imaging. Similarities of performance and brain function were more striking than differences between the two groups, but a number of differences were evident. During memory performance, normotensive participants showed a greater regional cerebral blood flow (rCBF) response in the posterior parietal and thalamic brain areas, while hypertensive participants showed a greater extent of activation in the prefrontal cortex [10]. Relatedly, the activation of different areas of the brain during the memory task was more highly correlated in hypertensive relative to normotensive participants [8]. Other investigators have reported relatively decreased grey matter volume in certain brain areas among hypertensive relative to normotensive individuals [11, 12], and we found differences as well [13]. Similarly, our results were consistent with prior reports of brain indices of aging. Namely, decreases in ventricular volumes in the brain as well as increases in prevalence of white matter hyperintensities were more evident in hypertensive individuals compared to similarly aged normotensive individuals, for example, [14]. Finally, blood pressure reactivity to laboratory challenges is known to prospectively relate to essential hypertension [15], and work of our colleague, Peter Gianaros, has shown consistent relationships between degree of activation in a number of limbic brain regions and degree of blood pressure response to challenges [16–19]. The results of our cross-sectional work clearly established that the human brain was impacted by hypertension. The presence of brain correlates of blood pressure responding in normotensive individuals suggested that blood pressure reactivity might be contributing to the later impact of hypertension on the brain. These results lead us to propose a vascular hypothesis suggesting that vascular responses as well as chronic cerebrovascular changes due to the disease had a subsequent impact on neural and thus neuropsychological (cognitive) function [7].

## 3. Longitudinal Findings

An intervention study based on our cerebrovascular hypothesis, however, led us to consider an alternative possibility that neural changes preceded or were concomitant with vascular changes induced by essential hypertension. Based on our vascular hypothesis, we treated previously unmedicated hypertensive patients for a year with an angiotensin converting enzyme (ACE) inhibitor or with *a beta* blocker. Prior work suggested that the ACE inhibitor would reverse vascular morphology changes associated with hypertension while the *beta* blocker would not, for example, [20, 21]. Remediation of the vascular morphology was then expected to enhance the capability of the hypertensive patients to vasodilate cortical vessels and hence more effectively adjust blood flow to active brain areas. In fact, despite excellent reductions in blood pressure by both medications, no differences between them or in pre- to postassessments occurred in resting or task-related cerebral blood flow [22]. Despite this failure of the test of our vascular hypothesis, some important observations were made. We had expected the lowering of blood pressure to reverse or stabilize brain indices that we had related to hypertension in our cross-sectional study. In fact, the degree of concomitant activation across brain areas increased and grey matter volume loss continued [23, 24]. Moreover, the degree of brain aging pretreatment and the robustness of the thalamic response to working memory pretreatment were predictive of the success of blood pressure lowering [22]. The state of the brain seemed to be a proxy for the severity of the essential hypertension. In short, a pathophysiological process underlying essential hypertension seemed to be continuing to influence the brain—peripheral pressure per se did not seem to be causing the brain changes that continued to be observed. Arguably, our intervention was only a year in the course of a long-lasting disease and alternative interpretations can be drawn. At present, we are attempting to support the hypothesis that brain changes are an early target of hypertension by examining whether brain signs of essential hypertension are present prior to or concurrently with the changes in blood pressure that lead to diagnosed essential hypertension.

## 4. Pathophysiology of Essential Hypertension

What are the implications of the possibility that essential hypertension is a disease that influences the brain early in its course? Furthermore, what factors might predispose individuals to essential hypertension and be so prevalent in our society that more than half of us over the age of sixty have essential hypertension? One implication is that factors influencing the brain rather directly, such as psychosocial factors, may be as important as biological and behavioral factors in contributing to the etiology of essential hypertension. This implication is hardly novel; it has been posited for many years, particularly within the psychosomatic medicine tradition. Few, however, believe that psychosocial factors are the sole cause of essential hypertension. The best concept of the causation of hypertension may still be the mosaic theory of Page [25, 26] that emphasizes the multiple factors known to influence the disease and the regulatory interactions between these factors. Essential hypertension is then expected to arise from not a single factor but rather numerous and likely different combinations of factors. Any particular combination of dysregulated processes may then overwhelm the complex, but redundant regulatory system that aims to maintain normal blood pressure. Approximately 30 years ago, Weiner [27] provided a comprehensive review of the psychobiology of hypertension with careful attention to extant work in humans and animal models. His approach echoed the mosaic theory, but evidence was updated and viewed from a psychosomatic perspective. The general conclusions from that review remain valid today. Essential hypertension appears to have multiple causes and may not even be a single disease. In addition, the factors inducing high blood pressure may not be the same as those maintaining the high blood pressure. The various etiological possibilities all involve a failure of regulatory function—within neural control of blood pressure, renal, and/or endocrine function.

Recent work has largely reinforced these conclusions [30–33]. Factors in the pathophysiology of hypertension noted by Page remain important and our knowledge of these individually are growing, for example, genetic, endocrine, and immune factors [5, 34–38]. Of note, greater attention to renal involvement in hypertension has been combined with greater knowledge of the interaction of renal and neural control [39–43].

We can examine the renin-angiotensin system more closely to illustrate the increasing importance of central regulation. The peripheral renin-angiotensin system has well-known influences on the kidney and vasculature, but maintenance of blood pressure also seems to involve renin-angiotensin present in the central nervous system. Angiotensin is present and appears active in the nucleus tractus solitarius and the dorsolateral ventral medulla—important blood pressure regulatory areas [44]. Circulating angiotensin is also known to influence the brain via transmission through the circumventricular organ. Brain angiotensin influences neuroendocrine systems and, more surprisingly, influences learning and memory in animal models [45, 46]. Finally, angiotensin is responsive to stress and antagonists appear to both lessen stress responses and improve learning and memory in animal models [45, 46]. Thus, a disruption of the renin-angiotensin system would concurrently impact both brain and vascular function. This may account in part for interest in medication influencing brain angiotensin receptors that preserves brain function while reducing peripheral blood pressure as well as other medication [47].

## 5. Central Factors in Essential Hypertension

As part of the mosaic of disease factors, the involvement of neural control in affected regulatory systems suggests that the nervous system is contributory to the etiology and maintenance of some, if not all forms of, essential hypertension as well as it being a target of the disease. In reviewing the then-popular concept of “borderline hypertension,” Weiner found stress to be a supportable factor influencing the pathophysiology of essential hypertension presumably through its impact on brain function. He found reasonably strong evidence for stressful experience moderating the development of hypertension in animal models, potential mediation by sympathetic nervous system dysfunction, and remediation of blood pressure with interventions on neural control. If anything since Weiner's review, psychological factors have been increasingly mentioned among initiating factors in concepts of hypertension unifying neural and renal factors in the disease, for example, [48, 49] The number of mechanisms which could be impacted by neural control has grown, but linking them to initiating factors is complex, particularly in humans. How the various influential factors become dysregulated and interact to create the natural history of regulatory failures that can lead to hypertension remains unknown. In short, as our knowledge of blood pressure regulation expands, the intertwining of neural, endocrine, and immune control becomes more evident as does the importance of central nervous system control. The robustness of these conclusions, however, should not be construed as resolving the primary question. We lack an empirically supported, general model of how blood pressure control is integrated such that essential hypertension occurs when a dysfunction occurs within one or, more likely, more contributory mechanisms.

 Coordination by the brain does, however, seem critical to the operation of such a multifaceted control system. Regulatory failure due to any cause must influence normal brain function because the brain normally maintains normotensive levels of blood pressure. Brain counterregulation must fail, the brain itself may be affected, or the range of regulation possible must simply be exceeded so that the brain becomes permissive of heightened pressures. From this perspective, the brain must be an early target of essential hypertension. It remains arguable whether brain pathology is a primary initiating factor in essential hypertension. What psychological/behavioral factors might impact the brain and contribute to hypertension and be consistent with the present day pandemic level of essential hypertension? Diet/salt intake, physical inactivity, and stress seem likely candidates—factors readily related to demonstrated influences on hypertension (see, e.g., [50]).

 For the remainder of our discussion we will focus on stress as an important factor that acts in concern with other biological and psychosocial factors. This focus is partially one of our personal interests, but one could argue that diet/salt and physical inactivity are themselves driven in part by coping with stress. In this context, it is important to point out that our discussion will proceed from accepting the global use of the term “stress” for purposes of the reviewing literature to a more specific examination of affective processing. In the future, it may be useful to assess separable influences of the environmental or psychological stressor, the individual's appraisal of such stressors, and their evaluation and use of particular coping strategies, see Lazarus [51, 52]. For example, the idea that overeating and physical inactivity are coping activities in response to stress is typically not well-supported empirically due to measurement difficulties as well as, typically, the investigators' greater interest in the maladaptive behavior itself and less with its exact source. Stress encompassing the constellation of involved stressors and processes provides a convenient proxy until we can analytically separate factors most critical for our dependent variable of interest, be it hypertension or dysphoria.

**Figure 1:**
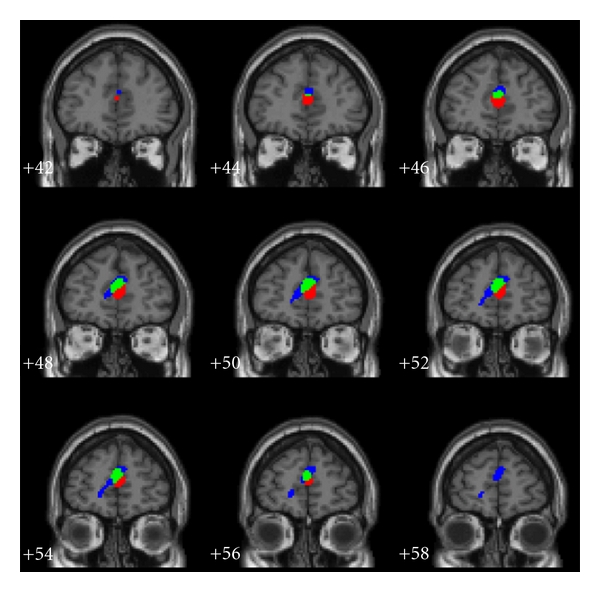
Dorsomedial prefrontal area in which greater regional cerebral blood flow post-treatment related to either better blood pressure decrease (red), poorer post-relative to pretreatment working memory performance (blue), or both (green). See details and relevant review [28, 29].

A second reason for our focus on stress is another finding from our treatment study that we briefly reviewed at the beginning of this paper. Examining individual differences in both the response to treatment of blood pressure and working memory performance, we observed a dorsomedial prefrontal area that was related to both [28]. The result suggested that activation in this area favored successful reduction in blood pressure levels but at the cost of poorer performance on working memory (in comparison to their pretreatment performance). As shown in Figure 1, in the same general brain area, we observed significant subareas in which significant regional cerebral blood flow activation posttreatment relative to pretreatment: (a) correlated with how well a participant had lowered blood pressure, (b) correlated negatively with the change in memory performance, and (c) correlated with both blood pressure lowering and memory performance—an area sharing these correlations. A thorough quantitative review of the literature [29] suggested that this virtually exact dorsomedial prefrontal area related to the contextual factors influencing emotion as well as to autonomic reactivity. Subsequent work (reviewed through http://www.neurosynth.org/) continues to support this functional relationship to affective responses to faces and scenes [53] and to autonomic nervous system responding [54, 55]. The latter paper is particularly relevant in that the dorsomedial prefrontal cortex showed an overlapping area that related to both autonomic responding (heart rate variability, blood pressure was not reported upon) as well as affective evaluation. Dorsomedial prefrontal cortex activation has been related to empathetic responses with greater activation related to less tendency for personal distress [56]; a finding related to the modulation of dorsomedial prefrontal activation when anticipatory set was used to modulate affect [57]. Notably though, some work has shown the dorsomedial prefrontal cortex to be related to greater cortisol response to stress [58]; while other work again suggests activation of this area to be related to lower sensitivity to fear and disgust [59]. Overall, the literature seems consistent with an interpretation that at least portions of the dorsomedial prefrontal area organize affective and autonomic response to a challenge. If so, we then speculate that successful blood pressure regulation altered this organization in our treatment study. After treatment, a damping of the cognitive/affective response to challenge may have been instrumental in maintaining lower blood pressure. If this interpretation is correct, we could suspect that individual differences in the affective evaluation and response to situations might relate to the control of blood pressure and that these individual differences that might be expected to relate to individual differences in activation of dorsomedial prefrontal cortex.

An alternative to examining affective regulation would be examining cognitive function vis-a-vis hypertension. Indeed, our work was launched based on reviews showing that hypertensive individuals performed slightly more poorly than normotensive individuals on tests of executive attention and working memory [9, 60]. Cognitive function, however, remained stable throughout our treatment study. In addition, regional cerebral blood flow responses to a working memory task were unaffected by the successful treatment of blood pressure.

## 6. Stress and Hypertension

The notion that stress causes hypertension is popularly accepted, but the scientific basis for this is somewhat lacking. An initial issue is simply that stress refers to a host of conditions so that the statement that stress causes hypertension is simply impossibly vague. Scientific usage varies between stress defined as a response to a laboratory computer challenge or a daily hassle, to stress defined as the loss of a spouse or as a response to a continuing divorce proceeding. The popularity of the term likely stems from the sense that external events rather than one's own personality or misbehavior are inducing a negative psychological reaction. Being stressed is more socially acceptable than other alternatives for explaining negative psychological states. Stress, though, is certainly an interaction between the person and the environmental situation as so elegantly described by Lazarus [51, 52].

Reviews of stress and hypertension vary somewhat in how they use the stress concept, but generally are only minimally supportive of a relationship between stress, variously defined, and hypertension. Nykliček et al. [61] found that objective measures of stress were related to heightened blood pressure (though not all studies assessed essential hypertension per se). Objective measures in this paper included exposure to natural disasters, unsafe neighborhood conditions, noise exposure, and stressful occupation. In contrast they report minimal association when self reported stress was related to blood pressure. They make a reasonable case for a difference between studies examining hypertensive patients aware of their disorder relative to studies examining individuals unaware of their blood pressure status. Hypertensive patients were seen as tending to report a stressful background, possibly as an explanation to themselves of their condition. Unaware individuals with high blood pressure were observed to report low levels of stress and overall high psychological wellbeing. The reviewers suggest that contradictory findings relating hypertension to recalled stressful life events and occupational stress may be due in part to this dependence of stress reporting on awareness of hypertension. Another review examines an individual difference dimension that was developed specifically in relation to stress and hypertension in African Americans. As reviewed by Bennett et al. [62], John Henryism refers to a style of coping with stress—specifically, continuous active coping—that theoretically would positively correlate with blood pressure in groups faced with chronic stress and few resources, that is, lower class African Americans. Initial evidence supported this conceptualization, but the review notes results suggesting that the concept is not specific to African Americans and has been related to blood pressure in some studies regardless of social class or even more strongly in upper classes. John Henryism is interesting since that coping style contrasts markedly with the earlier descriptions of the hypertensive personality (see below). The concept may align more closely with work stress in the presence of little job control. Nonetheless, the review suggests that the relationship of John Henryism to blood pressure is now more obscure than when the concept was introduced. A more recent review [63] selected only studies that were longitudinal or used case-control designs (reducing studies examined from 82 to 14, but still with over fifty-two thousand individuals included). Three studies examined the hypertensive aftermath of acute stressors, such as natural disasters, and no association was found between the acute stressors and blood pressure. This is in contrast to the prior review and suggests that the shorter term blood pressure increases after acute stress events may not be sustained, that is, essential hypertension may not be expressed after 3 years or more have passed since the acute event. When chronic stress was reviewed, the seven qualifying studies generally supported an association with better evidence for job stress. Affective response was examined and included reports of hopelessness and racial discrimination, that is, internalized responses assumed to result from a history of stressful events. Although mixed, the majority of these studies did show an association albeit not large between the affective response and hypertension. Overall, positive odds ratios from their reviews predominated, but the authors reserved judgment until better-designed studies with well-defined exposures and control for confounds were available. It is unfortunate that this review did not consider the hypertension awareness issue raised in the Nykliček review.

## 7. Cardiovascular Reactivity and Stress

One definition of stress of direct relevance to hypertension refers to the acute changes in blood pressure that occur in the laboratory in response to mental challenge tasks. Participants report that they find such tasks stressful, so the blood pressure responses to such tasks are then defined as a stress response. The mechanism of how such responses lead to essential hypertension is difficult to establish, although such responses do relate to the incidence of later hypertension [64], and, as noted above, brain mediation of such individual differences in responsivity seems established [16, 17, 65]. Reviews support the prospective relationship between heightened blood pressure response to such laboratory challenges and subsequent hypertension [15, 66]. This relationship has not been clearly shown to be mediated by psychological factors although there is a report of defensiveness contributing to the relationship between blood pressure reactivity to laboratory stress and subsequent high blood pressure [67]. The psychophysiological pathways between such acute pressure changes and the chronic disease state have been discussed by Krantz and Manuck [68]. As they note, the prospective correlation of acute blood pressure reactions and later hypertension might be seen as mechanistic if the laboratory reactivity generalized to either repetitive reactions in daily life or tonic increases during daytime or sleep hours [69, 70]. Strong evidence for such relationships has not been found, however, [64] although concepts of sustained reaction to acute events have been proposed to interact with other factors in the etiology of hypertension [71]. A promising, but not yet completely established, link would be between hypertension and delayed recovery of cardiovascular responses after an acute stressor as possibly related to continuing intrusive thoughts relevant to the stress, that is, rumination [72]. In short, stress defined as a cardiovascular reaction can be related to hypertension, but this defines stress in a circular fashion so our knowledge of psychological factors, more commonly thought to involve stress, is minimally advanced.

## 8. Pain and Hypertension

Although stress results in discomfort, the term is not usually applied to the acute reaction to a noxious stimulus, pain. Nonetheless, pain can be seen as a negative affect and pain has been well studied as a function of blood pressure. Pain perception is importantly related to blood pressure. France [73] reviewed the large body of evidence relating diminished pain sensitivity in animals and humans to high blood pressure. He also addressed the question of whether hypertension induces a reduction in pain sensitivity or if pain sensitivity changes precede the development of hypertension. Earlier, in a well-known paper, Dworkin et al. argued that acute blood pressure increases reduced pain and thus hypertension was acquired through instrumental learning reinforced by this reduction in pain sensitivity [74, 75]. Their model suggested that the baroreceptors in the carotid sinus and aortic arch responded to transiently increased pressure through transmission to the nucleus tractus solitarius, which then had a damping effect both on pain perception and blood pressure, see discussion of baroreceptor function in Berntson et al. [76]. The association of a reduction in pain contingent on an increase in blood pressure was the mechanism posited to lead to hypertension. This model has mixed empirical support and France [73] sought more general support for the hypothesis that pain hyposensitivity precedes the development of hypertension. This is supported by work in animal models of hypertension; at risk animals tested prior to the development of hypertension showing greater pain hyposensitivity relative to animals not at risk. Studies of humans at risk for hypertension due to family history and/or heightened resting blood pressure also have been observed to show a pain hyposensitivity. Finally, hypertension has been related to reduced pain reports during induction of baroreceptor reflexes. Based on this review, France [73] speculated that pathophysiology of the paraventricular area of the hypothalamus may be the mechanism for the reduced pain sensitivity. Functioning of this area is thought to act on pain through action of the baroreceptor reflex and its impact on enkephalins, through heightened opioid stimulation, or heightened activation of descending pathways inhibiting pain. Although exceptions exist, the opioid changes related to heightened blood pressure are the best known among these supposed pathways [77–79]. Recent work on visceral and somatic afferents, however, demonstrate surprisingly sustained effects on blood pressure of small fiber stimulation—effects mediated by long loop connections with the arcuate nucleus of the hypothalamus (known to regulate endogenous opioids) and with connections to neural areas known to exert sympathetic nervous system influences on blood pressure (i.e., rostral ventrolateral medulla) [80].

The relevance of this relationship between blood pressure and pain perception is increased by recent studies on both brain imaging and the perception of emotion. Recent brain imaging studies have examined the “pain” of social exclusion and observed a striking overlap between brain regions responsive to this and to physical pain [81, 82]. Such findings support observations suggesting that blood pressure regulation may be more generally related to affect, for example, [83]. Relatedly, hints of an empirical relationship between underreporting of affect/wellbeing and the relative insensitivity to pain often observed among hypertensives were also found by Nyklíček et al. [84].

## 9. Personality and Hypertension

The examination of “affective response” as related to stress renders another review relevant; one relating hypertension to psychological factors closer to personality in most cases [85]. This review relates to the long history of cross-sectional studies claiming to reveal a “hypertensive personality” (see [27, 86, 87]), but chose to examine only prospective, longitudinal studies excluding the possibility of an influence of “hypertension awareness” and more reasonably raising the possibility that the psychological factor have a causative role. Fifteen qualifying studies were found. The majority of these examined negative affect (anger, hostility, defensiveness), but depression, neuroticism, and psychopathology were also represented. Among these studies, the majority was observed to show a relationship, but the strength of the overall relationship was low—corresponding to an r of.08. The Sparrenberger review [63] comments that some subsequent studies after the date of this review specifically examining depression have not observed a relationship.

## 10. Psychological Processes and Hypertension

Although physiological reactions, recovery, and pain sensitivity could mold personality and behavior, it seems more likely that some aspect of personality might lead to consistent interactions with the environment creating a risk for hypertension. Such a personality feature might relate directly to hypertensive risk or might act through cardiovascular reactivity. (To date, however, a personality mediator for blood pressure reactivity results has not been evident.) We assume that this aspect of personality might contribute to the present, but small relationships observed between stress broadly conceived (as in the reviews above) and high blood pressure. Based on our brain imaging results and the close relationship between stress and affect, in the remainder of the paper we will focus on current developments relating to the question of whether a particular aspect of affect perception and expression may be important in the etiology or early stage of essential hypertension.

As noted earlier, early psychosomatic theories propose a “hypertensive personality” that contributes to the development of hypertension [1, 86–89], with persons at risk for hypertension showing increased emotionality in everyday situations [86, 87]. Other work suggested a blunted perception of negative events that might be yet more specific than a personality type [90]. In the late 1970s Weiner [27] reviewed this literature as suggesting a relatively socially withdrawn personality that tended to be avoidant, but that could be hostile. Subsequent literature focused on the negative affect aspect. As noted above, a review of studies prospectively relating psychological factors to the development of hypertension did observe a small, but quantitatively supported relationship between measures of negative affect and hypertension development [85]. This relationship showed little specificity, however, as the measures showing predictivity included anger-in, trait anger, anger-out, anxiety, depression, anger-control, defensiveness, hopelessness, neuroticism, hostility and psychopathology.

## 11. Affectivity and Hypertension

There is a large body of literature connecting affect and hypertension [27, 91, 92]. The concept of a “hypertensive personality” origin of hypertension failed to achieve solid support and research moved toward the belief that hypertension and affective changes result from a common etiology related to affect. The literature continued to relate elevated blood pressure and essential hypertension with lower affect expression, more negative affectivity, and defensiveness (for a comprehensive review that goes beyond that possible here, see Jorgensen et al. 1996 [91]). Of note is a review by [92] explicitly examining whether high negative affect (anger experience) combined with inhibited anger expression, that is, one version of the “hypertensive personality”, was related to blood pressure. They failed to find this relationship but did show a small quantitative relationship between experience of negative affect and blood pressure—anger expression was not consistently related to blood pressure [92]. Of interest though, they note that the two scales most strongly relating negative affect/anger experience to blood pressure assess both these feelings as well as a reluctance to express such feelings.

The reluctance to express or even recognize affect has been examined separately as a correlate of blood pressure. Research on alexithymia, the inability to describe one's emotions, and hypertension has shown a positive relationship. A number of studies using shortened versions of the Toronto Alexithymia Scale (TAS) have found higher scores on the TAS-20 or TAS-26 to be correlated with higher blood pressures [93–96]. None of these studies have however been able to move beyond the observed relationship to an explanation of how hypertension leads to alexithymia or vice versa. Furthermore, there is some disagreement about the general relationship of alexithymia and hypertension [97, 98] as well as concern that only particular aspects of alexithymia may relate to heightened blood pressure [99]. Alexithymia may be considered to reflect an inability to verbalize one's own emotions, but it might also reflect a general impairment in the ability to recognize emotion. Lane et al. [100], for example, found that two alexithymia measures, the Toronto Alexithymia Scale and the Levels of Emotional Awareness, both correlate with the Perception of Affect test, which is designed to assess accuracy of verbal, non-verbal, and mixed (verbal and nonverbal) emotion recognition. This relationship potentially links alexithymia to the literature on decreased emotion recognition in hypertensives.

Blunted perception of negative affect as well as blunted expression of affect once established has been suggested to help those unsuccessful at interpersonal conflict to avoid these situations resulting in a “profile of submissiveness, conflict avoidance, and low levels of anger experience and expression” [91]. A review by Jorgensen et al. focused on studies looking at measures of affect expression, negative affectivity, defensiveness (characterized by denial, repressive coping, and damping of affective function), or a combination of these in conjunction with measures of blood pressure [91]. The resulting meta-analysis of 83 studies found that individuals with higher blood pressure tended to have lower affect expression but higher negative affectivity and defensiveness than those with lower BP [91]. Additionally, awareness of hypertensive status was a significant predictor of study outcomes for both affect expression and negative affectivity [91]. Studies were not available to test whether awareness of hypertensive status influenced defensiveness. Of note, whether they were aware or unaware of their blood pressure levels, participants with high blood pressure were lower on affect expression than participants with lower blood pressure levels. Ultimately the analysis found defensiveness to be the most robust predictor of high blood pressure [91]. The authors speculated that defensiveness may be linked to the pain/opiod system: “(This association is) consistent with the involvement of central opioid-peptide mechanisms in the covariation of essential hypertension with defensiveness” [91, p. 311].

Affect might not be the central concept–an alternative perspective posits that hypertension induced changes in cognitive processing (resulting in deficits in perception, processing and recall of information) impairs affective responses to demanding interpersonal interactions [101]. Cognitive deficits have, however, proven to be relatively subtle and not present in low level processes such as perception, speed of processing, or even long-term memory [9, 60]. Mild deficits or blunted recognition and expression of emotion might, however, lead to a history of unsuccessful interpersonal interactions and through this to low self-efficacy and avoidance behavior [102, 103].

## 12. Affect and Pain

Other research has focused on affect as a mediator of pain perception in hypertension. An understanding that the perception of pain is not simply a physical response led researchers to explore the connection between hypoalgesia, affect, and hypertension. Fillingim and colleagues suggested that the effect of elevated blood pressure on pain is the result of reduced affective response rather than reduced pain sensitivity [104]. This relates to the surprising and reasonably consistent finding that negative events and symptoms are reported as lower among those with higher blood pressure, but only among those not diagnosed with hypertension [61, 84, 105]. This relationship does not seem to be necessarily related to negative affect/anxiety/defensiveness [105], but as noted above may be related to pain perception [105]. Associating possible underlying mechanisms, Wilkinson and France speculated that it is possible that changes in sensory, affective, and cognitive processing of noxious stimuli may influence hypoalgesia in hypertensives or those who are at risk for hypertension [106]. They looked at the activation of baroreflexes in conjunction with response to positive, negative and neutral affective stimuli. Though no connection was found between baroreflex stimulation and affective measures, an interaction between parental history of hypertension and mood showed reduced emotional valence ratings for both positive and negative images, but not for neutral images for subjects who had a positive parental hypertension history versus those whose parents did not have hypertension [106]. For ratings of arousal, those who have a positive parental history of hypertension reported less arousal to both positive and negative stimuli and higher arousal to neutral stimuli when compared to participants who had normotensive parents [106]. Although this study did not find support for the role of baroreflex stimulation in affective responding among individuals at risk for hypertension, the discovery of dampened emotional responses to both positive and negative stimuli in conjunction with similarly dampened autonomic and involuntary measures of response (i.e., skin conductance and EMG) for participants with a parental history of hypertension reinforce the belief that a common underlying mechanism contributes to the changes in affect, blood pressure, and other autonomic responses [106]. 

## 13. Positive Affect

It is important to keep in mind that affective response is multidimensional (e.g., valence positive-to-negative state and arousal high-to-low energy state) as a result, expansion of research into positive affectivity seemed the obvious next step. Pury et al. proposed that emotional responses of persons with high blood pressure to a broader range of stimuli (things other than stress and physical pain) can happen in 3 different ways: (1) lessened response to negative stimuli with no change in response to positive stimuli, (2) more positive responses to both positive and negative stimuli, or (3) “dampened emotional responses” to both positive and negative stimuli [107]. In their study Pury and colleagues took resting blood pressures of 65 normotensive young adults and then asked them to rate a series of photographs on valence and arousal [107]. Systolic BP was found to be associated with more neutral ratings for all of the photos, as well as for the positive photos and for the negative photos [107]. These findings show a dampened emotional response to visual stimuli for persons with a higher resting SBP causing Pury and colleagues to suggest that “prior research on the relationship between blood pressure and subjective ratings of negative stimuli (pain, psychosocial stress) may be reflecting a general tendency toward reduced responsiveness to emotionally provocative stimuli in general” [107, p. 585]. A more recent study by McCubbin et al. [108] found that zero order correlations show an inverse relationship between the Perception of Affect task scores and both systolic and diastolic blood pressure in a predominantly African American sample. This finding further supports the theory of emotional dampening in persons with elevated blood pressure [108]. In summary the current literature seems to point at an evolving relationship between hypertension and affectivity. With those likely to develop hypertension exhibiting intense emotionality specifically in relation to the expression of anger and developing into blunted perception and expression of both positive and negative emotion [86, 87, 91, 107]. The evolution in the relationship between hypertension and affect further supports the idea for a common etiology, however there is much yet to be explored.

## 14. Concluding Remarks

Continued exploration into both positive and negative aspects of emotionality will be important in understanding the connection between hypertension and affectivity. Additionally, understanding this psychosomatic relationship requires careful concurrent assessment of both physiology and psychological function using valid and reliable measures. As Suls et al. [92] note, earlier work has been characterized by either careful psychological or physiological assessment but rarely by care in both areas. Prospective studies of the development of hypertension while monitoring affect and cognition will add much to the existing literature. As the relationship of blunted perception of affect (or of negative events) continues to be explored it should be useful to relate it to concepts of the development of hypertension. Weiner [27] reviewed work suggesting that normal blood pressure advanced to hypertension through a prehypertensive phase that could take different forms: initial increase in cardiac output, an increase in both heart rate or cardiac output, or an increase in peripheral vascular resistance. Although work has not refined these speculations greatly, the point is that the hypertension likely results from a variety of mechanisms and one of more of these may relate more strongly to the perceptual sensitivity characterizing some individuals that advance to hypertension.  Table 1 is a rough table of what is somewhat known about physiology, affect, and cognition at different stages of hypertension. The table is incomplete, particularly in terms of the multiple physiological mechanisms suspected to impact hypertension. Brain indices are included but very little work has been done in this area. We have tried to develop the case that brain indices as well as self-report indices are pointing to a possible convergence that may enlighten us on how exactly affect, its perception, regulation, and expression may be related to the natural history of hypertension. As research goes forward, assessment of both continuous blood pressure as well as categorical measures relating to medical definitions of the disease should be included with attention to the issue of whether individuals are aware or unaware of their hypertensive status. The area would further benefit from the use of well-developed scales of affect that can be compared across studies as well as the use of any newly developed measures that capture hypotheses about the tantalizing relationship between affect and hypertension.

**Notes**

Progression of blood pressure with age derived from Franklin and Mitchell [109], early neuropsychological deficits [110, 111]. Physiological characterizations are incomplete; borderline/prehypertensive based on Köhler, Fricke, Ritz, & Scherbaum et al. [112]. Alzheimer relationship based on Wu et al. [113]. Dysregulation in multiple systems may independently lead to hypertension and the timing of such changes are largely unknown in humans. Evidence for this can be readily deduced from the variety of animal models that achieve hypertension via different routes [114].

## Figures and Tables

**Table 1:** Diagrammatic illustration of integrated study of the natural history of hypertension.

Stage of Disease	Physiology	Affective	Cognitive
Normotensive/youth (genetic/familial risk)	All normal possible hyper-reactivity to lab stress, Mild elevation in SBP Opioid dysregulation	Anger? Reduced pain sensitivity	Subtle spatial attention, short term memory deficit
Borderline/Pre-hypertensive	BP >119/79 <140/90 with predominance of elevated DBP, sympathetic activation High cardiac output Baroreceptor adjustment; hyper reactivity to lab stress	Interpersonal difficulty Pain insensitivity Less awareness negative affect, positive affect?	
Early Hypertension (40–60yrs)	BP >140/90 High TPR Salt/diet sensitive Renin/angiotensin Aldosterone Sympathetic Structural/function brain changes Hyperreactivity	Above with transition to greater negative affect with inhibition of the expression of intense angry cognitive and emotive reactions	Mild deficits executive attention, working memory
Late Hypertension (60+ yrs)	Same BP or isolated systolic hypertension? maintenance of altered regulatory system	Continued high negative affect and expression of negative affect?; awareness of BP status may invert relationship	Deficits not as clear relative to age matched; Related to Alzheimer's Disease
